# CircRNA8220 Sponges MiR-8516 to Regulate Cell Viability and Milk Synthesis via Ras/MEK/ERK and PI3K/AKT/mTOR Pathways in Goat Mammary Epithelial Cells

**DOI:** 10.3390/ani10081347

**Published:** 2020-08-04

**Authors:** Chao Zhu, Yue Jiang, Junru Zhu, Yonglong He, Hao Yin, Quyu Duan, Lei Zhang, Binyun Cao, Xiaopeng An

**Affiliations:** College of Animal Science and Technology, Northwest A&F University, Yangling 712100, Shaanxi, China; chaozhu5739@163.com (C.Z.); jyue2019@163.com (Y.J.); zjr18710872378@163.com (J.Z.); heyonglong@nwafu.edu.cn (Y.H.); yh18392532130@163.com (H.Y.); 18291902995@163.com (Q.D.); zhanglei07dongke@163.com (L.Z.); caobinyun@126.com (B.C.)

**Keywords:** circRNA8220, miR-8516, *STC2*, β-casein, triglycerides

## Abstract

**Simple Summary:**

Yield and quality of goat milk are important indexes for screening dairy goat breeds. Therefore, it is necessary for us to improve the yield and quality of goat milk. In this study, we demonstrated that circRNA8220/miR-8516/*STC2* could promote the synthesis of β-casein and triglyceride through PI3K/AKT/mTOR pathway. In addition, we found that circRNA8220/miR-8516/*STC2* also promote proliferation via Ras/MEK/ERK pathway in goat mammary epithelial cells (GMECs). These findings contribute to a better understanding of circRNA-controlled breast development and lactation mechanisms and provide new potential insights into the regulation of breast development and milk composition in dairy goats.

**Abstract:**

Circular RNAs (circRNAs), which are considered a large class of endogenous noncoding RNAs, function as regulators in various biological procedures. In this study, the function and molecular mechanisms of circRNA8220 in goat mammary epithelial cells (GMECs) were explored. CircRNA8220 could spong miR-8516 and block the function of miR-8516 by binding to the target site of miR-8516 a negative feedback relationship existed between circRNA8220 and miR-8516. Stanniocalcin 2 (*STC2*) was a target gene of miR-8516. circRNA8220 could up-regulate the expression of *STC2* by sponging miR-8516 in GMECs. circRNA8220/miR-8516/*STC2* could promote proliferation and enhance the synthesis of β-casein and triglycerides (TG) via Ras/MEK/ERK and PI3K/AKT/mTOR signaling pathways, respectively.

## 1. Introduction

Mammary epithelial cells are mammalian secretory cells, which are the basis of lactation in goat mammary gland. The number and activity of mammary epithelial cells are closely related to breast lactation and play an important role in breast development [1]. Hypoplasia of mammary gland leads to decrease of milk production result in economic losses [2]. Therefore, a better understanding of molecular mechanisms underlying the development of mammary epithelial cells for increasing milk production is critical. However, the underlying molecular mechanisms of lactation in dairy goats is not clear.

Circular RNAs (circRNAs), which are RNA molecules that are characterised by covalently closed loop structures, down-regulate gene expression at the transcriptional or post-transcriptional level by binding to miRNAs or other molecules [3]. For example, circRNA_100290 regulates CDK6 expression by sponging miR-29b family members [4]. Hsa_circRNA_103809 promotes the expression of ZNF121 by sponging miR-4302 in lung cancer cells [5]. To date, many functions of circRNA have been found; for example, circRNA_100782 plays a role in proliferation regulation of pancreatic carcinoma [6]. Antagonistic roles of circRNA_100338 and miR-141-3p have been found in the regulation of invasive potential in liver cancer cells [7]. We selected circRNA8220 from circRNA libraries that were constructed by our team through bioinformatics prediction approach. However, the regulatory mechanism of circRNA8220 on milk synthesis and proliferation in goat mammary epithelial cells (GMECs) are still unclear.

miRNAs, which are noncoding single-stranded RNA molecules, regulate gene expression by binding to the 3′-untranslated region (UTRs), 5′-UTRs and even coding sequences of their target mRNAs; then, they affect a series of physiological functions in animals [8,9,10]. Incremental proofs demonstrate that miRNAs have an important regulatory function on milk synthesis [11,12]. The expression of miR-8516 has been found to be significantly different in peak lactation compared with prelactation [13]. However, no research on miR-8516 and effect of miR-8516 is available. Thus, studying the function of target gene of miR-8516 in GMECs is interesting.

Stanniocalcin 2 (*STC2*), which is a glycoprotein hormone, regulates a series of biological processes in an autocrine or paracrine manner [14], such as cell proliferation [15], tumorigenesis [16] and atherosclerosis [17]. *STC2* promotes colorectal cancer development and epithelial–mesenchymal transition process via activating MEK/ERK and PI3K/AKT signaling pathways [16]. However, the function of *STC2* in milk synthesis, proliferation and apoptosis of GMECs is unknown.

On the basis of these considerations, we decided to explore the function and mechanism of circRNA8220 and *STC2* in GMECs in vitro. Our findings revealed that circ-8220 could bind to miR-8516 via the target site inhibit miR-8516 activity lead to increase of *STC2* expression, promote GMECs proliferation and enhance the synthesis of β-casein and triglycerides (TG) via Ras/MEK/ERK and PI3K/AKT/mTOR signaling pathways, respectively. In addition, this study explored the effects of circ-8220 and *STC2* on proliferation and lactation in vitro.

## 2. Materials and Methods

### 2.1. Animals and Cell Culture

The Guanzhong dairy goats were selected from Longxian Goat Breeding Center near Northwest A&F University of China. Breast tissues were collected periods from three healthy Guanzhong dairy goats (3 year olds) at the peak of lactation by surgery. First of all, one side of the mammary gland of the dairy goat was wiped clean with alcohol then local was performed on the mammary tissue. A 5–6-cm incision was made and the skin pulled back using sterile forceps, exposing the mammary tissue. 1 cm^2^ samples were taken using a sterile scalpel blade and forceps. In order to stop any external bleeding, pressure was applied with sterile gauze to stop any external bleeding. The incision was closed with 6 to 8 surgical staples (#89063337, Appose ULC Skin Stapler, 35 wide; Henry Schein Inc., Melville, NY, USA). Mammary gland tissue was stored in PBS, added with 100 μg/mL streptomycin and 100 μg/mL penicillin and transferred to the laboratory within 1 h. The GMECs were cultivated in DMEM/F12 medium (Gibco, CA, USA) containing 10% foetal bovine serum, 10 ng/mL epidermal growth factor 1 (EGF-1, Gibco, CA, USA), 5 mg/mL insulin, 100 U/mL streptomycin/mL penicillin and 0.3 mmol/L hydrocortisone at 37 °C in a humidified atmosphere with 5% CO_2_. Human embryonic kidney cell line (HEK293T) was cultured in high-glucose DMEM (Dulbecco’s Modified Eagle Medium) supplemented with 10% fetal bovine serum, 100 μg/mL penicillin and 100 μg/mL streptomycin at 37 °C and 5% CO_2_ to proliferate. The GMECs were purified and cultured depending on previous reports [18].

### 2.2. pcDNA3.1-STC2 Vector Construction

Goat *STC2* CDs sequence (XM_005694539.3) was obtained by PCR using total RNA extract from GMECs, inserted into pMD™19-T vector (TaKaRa, Beijing, China) and entirely sequenced. Thereafter, *STC2* CD sequence was inserted into pcDNA3.1 vector (Thermo Fisher, Shanghai, China) between the *Xho I* and *Kpn I* sites. The insertion of the sequence into pcDNA3.1 vector was checked by entire sequencing. The forward primer of *STC2* was (*Hind III*) 5′-CTTAAGCTTATGTGTGCCGAGCGGCTG-3′ the reverse primer was (*Not I*) 5′-CGAGCGGCCGCTCACCTCCGGATATCGGAATACTCA-3′.

### 2.3. pCD5-circRNA8220 Vector Construction

Goat circRNA8220 full length was obtained by PCR and inserted into pMD™19-T vector. Thereafter, the sequence was subcloned into the pCD5-ciR vector (Geneseed, Guangzhou, China) between *EcoRI* and *BamHI* sites. The forward primer of circRNA8220 was (*EcoRI*) 5′-CGGAATTCTAATACTTTCAGCTGGAGCTGATTGGACACAAT-3′ the reverse primer was (*BamHI*) 5′-CGGGATCCAGTTGTTCTTACCTCTCATCACAGTAGGTGAAC-3′.

### 2.4. Transfection and RT-qPCR

**Figure A1:**
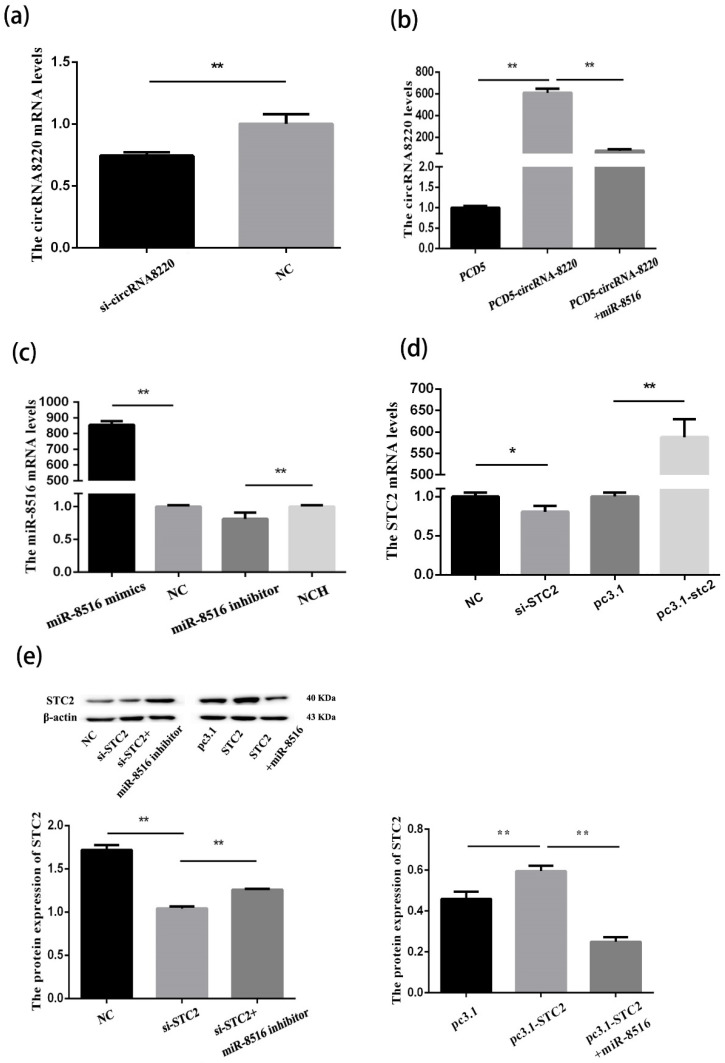
Transfection efficiency of pcDNA3.1-STC2 and pCD5-circRNA8220 vectors and STC2-siRNA, circRNA8220-siRNA and miR-8516 mimics/inhibitors. (**a**,**b**) mRNA levels of circRNA8220. (**c**) mRNA levels of miR-8516. (**d**) The mRNA levels of STC2. (**e**) Protein levels of STC2. ** *p* < 0.01, * *p* < 0.05.

When GMEC density reached 70–80%, pcDNA3.1-STC2 and pCD5-circRNA8220 vectors, STC2-siRNA, circRNA8220-siRNA and miR-8516 mimics/inhibitors and NC and inhibitor NC (GenePharma) were transfected using Lipofectamine™ RNAiMAX Reagent (Invitrogen, CA, USA) following the specifications of the manufacturer. At 24 or 48 h later, total RNA was extracted using Trizol reagent (TaKaRa, Dalin, China) and converted to cDNA using the Prime Script RT reagent kit with gDNA eraser (TaKaRa, Dalin, China) in accordance with the specifications of the manufacturer. The mRNA concentrations of interest were quantified via SYBR Premix Ex Taq II (TaKaRa, Beijing, China). The results were acquired from the CFX Connect Real-Time PCR Detection System (Bio-Rad, CA, USA). The PCR reaction procedure was as follows: 95 °C for 10 min 40 cycles at 94 °C for 15 s, 60 °C for 30 s and 72 °C for 30 s. Gene expression compared with β-actin or U6 mRNA level was calculated by the 2−^ΔΔCt^ method. All primers for RT-qPCR are shown in Appendix A Table A1. The transfection efficiencies of pcDNA3.1-STC2 and pCD5-circRNA8220 vectors and STC2-siRNA, circRNA8220-siRNA and miR-8516 mimics/inhibitors (GenePharma) are shown in Appendix A Figure A1.

### 2.5. Luciferase Reporter Assay

To explore miR-8516 whether target gene *STC2* and circRNA8220, 260 bp sequence of *STC2* 3′UTR and 344 bp total sequence of circRNA8220 were cloned into the psiCHECK-2 vectors (Addgene, CA, USA), respectively. The mutated plasmids with mutated target site (psiCHECK-2-Mut) were also constructed. The wild-type (psiCHECK-2-WT) or mutated (psiCHECK-2-Mut) plasmids were co-transfected with miR-8516 mimic or inhibitor into 293T cells using Lipofectamine™ RNAiMAX Reagent. At 24 h later, the result was obtained from thermo scientific varioskan flash (Thermo scientific, USA) using the Dual-Glo luciferase system (Promega, USA). The primer sequences are shown in Appendix A Table A1. Each experiment was performed three times in triplicate.

### 2.6. Cell Apoptosis and Proliferation Assay

The apoptosis ratio of GMECs was detected using Annexin V-FITC PI staining apoptosis assay kit (SeaBiotech, Shanghai, China) by flow cytometry method (FCM) after 24 h transfection following the protocol of the manufacturer. The viability of GMECs was tested by CCK8 assay. A total of 10 μL CCK8 solution (ZETA^TM^ life, USA) was put in each well after 24 h transfection. The resultant was then incubated in 96-well plates at 37 °C for 2 h. The results were calculated at 450 nm using an epoch microplate reader (Biotek, Winooski, USA).

To prove the credibility of the proliferation assay results in GMECs, the 5-Ethynyl-2′-deoxyuridine (Edu) assay was implemented in 96-well plates after GMECs were treated for 24 h. The cells were then washed by PBS three times after dyeing with Edu (Ribobio, Guangzhou, China) with a final concentration of 50 μM for 2 h. Thereafter, the cells were dyed with DAPI for 15 min in 37 °C. The result was observed by fluorescence microscopy.

### 2.7. Detecting the Concentration of β-casein and TG

Supernatant of cell culture medium and the cells were collected after 24 h transfection. The concentrations of β-casein and TG (triglyceride) were detected using Goat β-casein ELISA (Enzyme-linked immunosorbent assay) KIT (MLBIO, Shanghai, China) and Goat TG ELISA KIT (MLBIO, Shanghai, China) in accordance with the instructions of the manufacturer. The results were obtained from an epoch microplate reader (Biotek, Winooski, USA) at 450 nm. Concentrations were calculated in accordance with the standard curve. Variable coefficient values of intra- and inter-assay were less than 15% and 10%, respectively.

### 2.8. Western Blot

Proteins were extracted from cells by adding RIPA (Radio Immunoprecipitation Assay) lysis buffer (Biotek, Beijing, China) and mixed with phenylmethanesulfonyl fluoride (PMSF, Solarbio, Beijing, China) at 0.1 mg/mL after 48 h post-transfection. The concentration of proteins (GMECs) was detected with the BCA (Bicinchoninic Acid) protein assay kit (Vazyme Biotech, Nanjing, China) approximately 30 μg total protein was added a 12% SDS–PAGE (polyacrylamide gelelectrophoresis). The separated proteins were transferred onto polyvinylidene difluoride membrane (PVDF, Merck Millipore, MA, USA). The polyvinylidene difluoride membrane was immersed in 10% skimmed milk powder and diluted with Tris-buffered saline inclusive of 0.1% Tween 20 (pH 7.6) for 2.5 h at room temperature. The membrane was immersed in corresponding primary antibodies overnight at 4 °C (Table 1). In the next step, the membrane was incubated in suitable HRP (horseradish peroxidases)-conjugated secondary antibodies against mouse, rabbit at 4 °C for 2 h. Proteins were visualised using ECL prime western blotting detection reagent (Amersham, GE Healthcare Lifesciences) by gel documentation system (Biospectrum 410, UVP). Proteins were quantified by the Quantity One program (Bio-Rad, CA, USA).

### 2.9. Statistical Analysis

Each experiment was repeated at least three times in biological and technical repeats. All the data were processed by SPSS 19.0 (Beijing, China). The results were shown as means ± SE (standard error) the differences were compared by one-way ANOVA (** *p* < 0.01, * *p* < 0.05).

## 3. Results

### 3.1. STC2 Was a Target Gene of miR-8516 in GMECs

**Figure 1:**
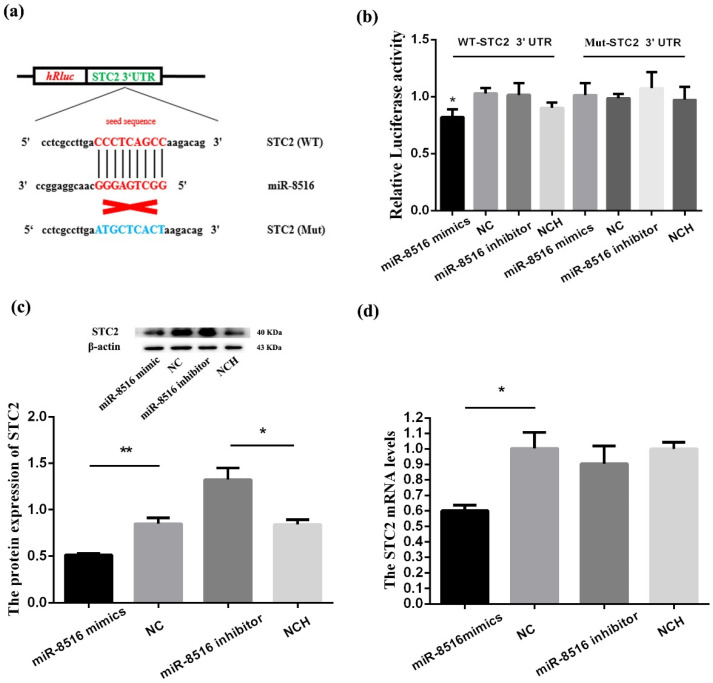
MiR-8516 down-regulated the expression level of STC2 via the 3′UTR. (**a**) The seed sequence of miR-8516 could match with WT-STC2-3′UTR and could not match with Mut-STC2-3′UTR. (**b**) Luciferase reporter assay of 293T cells co-transfected with WT-STC2-3′UTR or Mut-STC2-3′UTR and miR-8516 mimic, NC (negative control), miR-8516 inhibitor or NCH (NC-inhibitor). (**c**) miR-8516 decreased STC2 protein level in GMECs. (**d**) miR-8516 decreased STC2 mRNA level in GMECs. ** *p* < 0.01, * *p* < 0.05.

TargetScan database (targetscan.org) was used to select a target gene of miR-8516 in GMECs. We chose wild-type (WT) STC2-3′UTR or mutant (MUT)-STC2-3′UTR to set up a two-tier luciferase assay with psiCHECK-2 vector. The results showed that the relative luciferase activities of co-transfection with miR-8516 mimics and psC-STC2-3′UTR-WT were significantly attenuated compared with co-transfection with NC and psC-STC2-3′UTR-WT; by contrast, no change was observed in the psC-STC2-3′UTR-MUT (Figure 1a,b).

Western blot and RT-PCR (Quantitative real-time polymerase chain reaction) were used to detect the influence of miR-8516 on mRNA and proteins of *STC2*. The expression levels of miR-8516 were significantly increased or decreased by transfecting miR-8516 mimic or miR-8516 inhibitor (Appendix A Figure A1). The results showed that overexpression of miR-8516 significantly decreased mRNA and proteins levels of *STC2* down-regulation of the expression of miR-8516 increased the proteins levels of *STC2* (Figure 1c,d). In summary, these results proved that miR-8516 acts as a demotivated regulator of *STC2* by directly binding to 3′UTR in GMECs in vitro.

### 3.2. CircRNA8220 Acted as a Sponge for miR-8516

**Figure 2:**
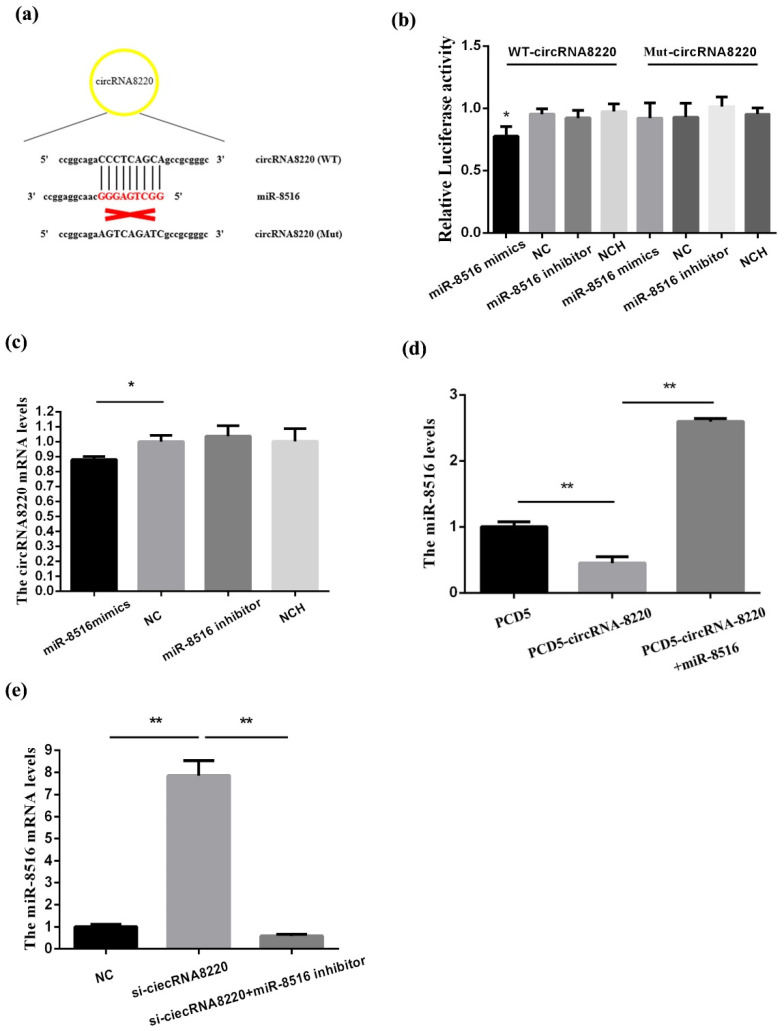
circRNA8220 acted as a sponge for miR-8516. (**a**) The seed sequence of miR-8516 could match with WT-circRNA8220 and could not match with Mut-circRNA8220. (**b**) Luciferase reporter assay of 293T cells co-transfected with wild type (WT)-circRNA8220 or Mut-circRNA8220 and miR-8516 mimic, NC (negative control), miR-8516 inhibitor or NCH (NC-inhibitor). (**c**) miRNA-8516 inhibited the mRNA expression of circRNA8220. (**d**) circRNA8220 decreased miR-8516 mRNA level in GMEC. (**e**) si-circRNA8220 promoted the mRNA expression of circRNA8220. ** *p* < 0.01, * *p* < 0.05.

miR-8516 seed sequence could match with circRNA8220 (Figure 2a). To verify this prediction, dual-luciferase reporter vectors were structured the luciferase activity reduced after co-transfection WT-circRNA8220 and miRNA-8516 mimic. However, the luciferase activity did not change after co-transfection MT-circRNA8220 and miRNA-8516 mimic (Figure 2b). We found that up-regulation of miRNA-8516 could decrease the mRNA levels of circRNA8220 in GMECs (Figure 2c). Overexpression of circRNA8220 decreased the mRNA levels of miR-8516 (Figure 2d). The level of miRNA-8516 was recovered after co-transfection pCD5-circRNA8220 vector and miRNA-8516. miR-8516 increased after silencing the expression of circRNA8220 using small interfering RNAs (siRNAs) the result was recovered by co-transfection si-circRNA8220 and miR-8516 inhibitor (Figure 2e). In summary, these findings proved that circRNA8220 could serve as a sponge for miR-8516 a negative regulatory relationship existed between circRNA8220 and miR-8516 in GMECs.

### 3.3. CircRNA8220 Increased the Expression of STC2 in GMECs In Vitro

**Figure 3:**
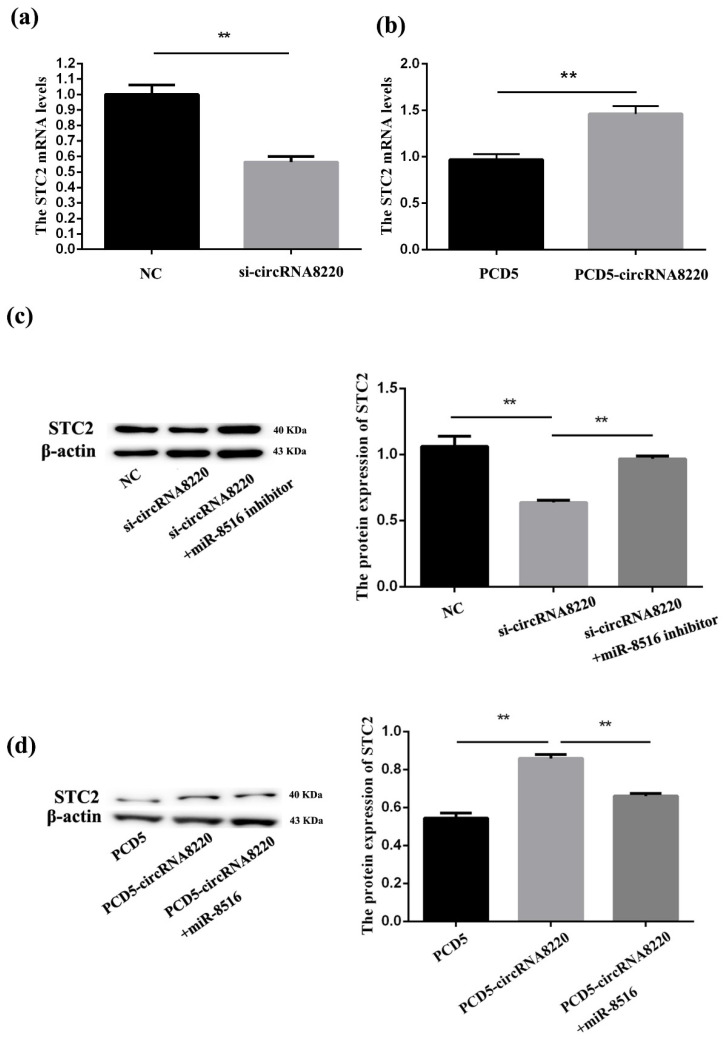
circRNA8220 increased the expression of STC2 in GMEC in vitro. (**a**) si-circRNA8220 reduced the mRNA expression of STC2. (**b**) circRNA8220 increased the mRNA expression of STC2. (**c**) si-circRNA8220 blocked the protein expression of STC2. (**d**) circRNA8220 raised the protein expression of STC2. ** *p* < 0.01.

qPCR was used to explore the impact of circRNA8220 on *STC2* in GMECs the results showed the mRNA level *STC2* was significantly down- or up-regulated after transfection of si-circRNA8220 or pCD5-circRNA8220. Western blot was also used to explore the impact of circRNA8220 on *STC2* in GMECs. We found that the protein level of *STC2* was down- or up-regulated after transfection of si-circRNA8220 or pCD5-circRNA8220; the mRNA and protein level of *STC2* were consistent with those of circRNA8220 (Figure 3). These results revealed that circRNA8220 could positively regulate mRNA and protein levels of *STC2*.

### 3.4. STC2 Inhibited Apoptosis and Promoted Proliferation of GMECs In Vitro

**Figure 4:**
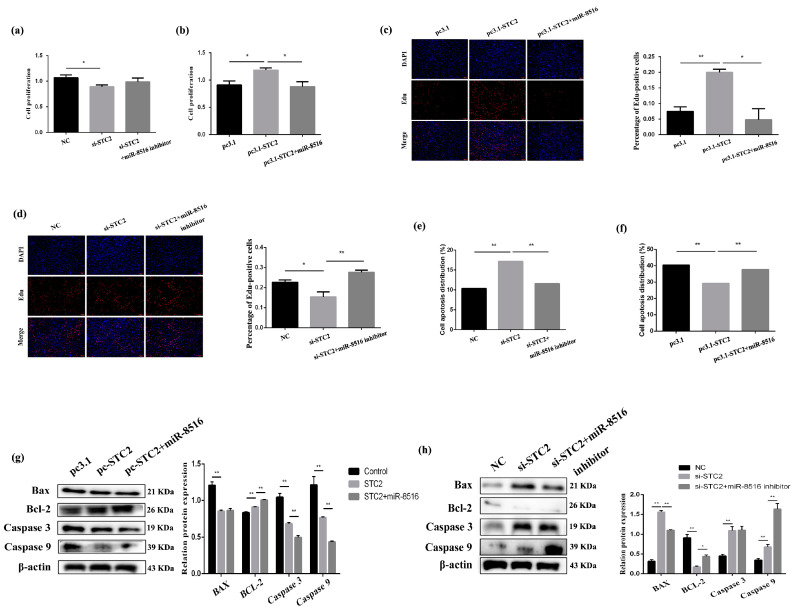
STC2 inhibited apoptosis and promoted proliferation of GMEC in vitro. (**a**,**b**) Cell proliferation was assessed using the cell counting kit-8 (CCK-8) assay after transfection with pc3.1-STC2 or si-STC2. (**c**,**d**) Cell proliferation indices were assessed after treatment with Edu after transfection with pc3.1-STC2 or si-STC2. (**e**,**f**) Apoptosis analysis of GMEC was detected with flow cytometry method after transfection with pc3.1-STC2 or si-STC2. (**g**,**h**) Protein levels of Bcl-2, Bax, caspase 3 and caspase 9 in GMEC after transfection with pc3.1-STC2 or si-STC2. Protein levels were measured by WB (western blot) densitometry was normalised to the β-actin density from the same lane. Data were expressed as the means ± SEM. ** *p* < 0.01, * *p* < 0.05.

STC2-siRNA and pcDNA3.1-STC2 vector were synthesized to study the effect of *STC2* on GMECs. The CCK8 assay was applied to explore the effect of viability of *STC2* on GMECs after transfection for 24 h. The data revealed that overexpression or inhibition of *STC2* enhanced or attenuated the viability of GMECs (Figure 4a,b). The Edu assay was conducted to verify the credibility of the proliferation assay results in GMECs; the result of the Edu assay was consistent with that of the CCK8 assay (Figure 4c,d).

Apoptosis ratios of GMECs after transfection with STC2-siRNA/pcDNA3.1-STC2 and co-transfection of STC2-siRNA with miR-8516 inhibitor/pcDNA3.1-STC2 with miR-8516 mimic were detected by Annexin V-FITC (fluoresceine isothiocyanate)/PI (propidium iodide) staining. The results showed that the rise or drop in the expression of *STC2* could attenuate or enhance the apoptosis ratio of GMECs (Figure 4e,f). The protein levels of critical apoptotic genes, including Bcl-2, Bax, caspase 9 and caspase 3, were also researched. We found that Bax, caspase 9 and caspase 3 all decreased significantly Bcl-2 increased (*p* < 0.01) after transfection with pcDNA3.1-STC2 after 48 h; the result was opposite when transfected with STC2-siRNA compared with pcDNA3.1-STC2 (Figure 4g,h). As expected, Western blot showed that miR-8516 mimic or inhibitor co-transfection with pcDNA3.1-STC2 or STC2-siRNA could partially weaken the effect of pcDNA3.1-STC2 or STC2-siRNA on apoptosis and viability. CCK8 and Edu in GMECs proved that miR-8516 could negatively regulate *STC2*. These consequences suggested that *STC2* could decrease GMEC apoptosis and induce proliferation in vitro.

### 3.5. Effect of circRNA8220 Was in Accordance with STC2 on Apoptosis and Proliferation of GMECs In Vitro

**Figure 5:**
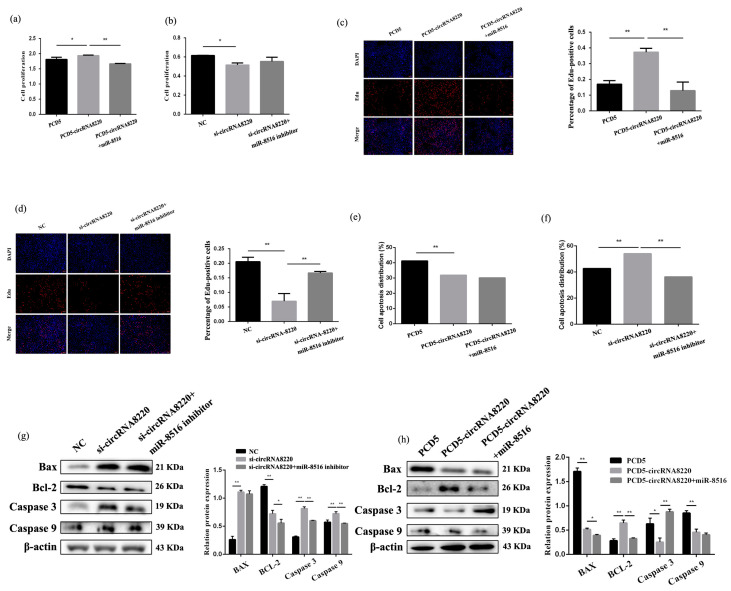
circRNA8220 inhibited apoptosis and promoted proliferation of GMEC in vitro. (**a**,**b**) Cell proliferation was assessed using the cell counting kit-8 (CCK-8) assay after transfection with PCD5-circRNA8220 or si-circRNA8220. (**c**,**d**) Cell proliferation indices were assessed after treatment with Edu after transfection with PCD5-circRNA8220 or si-circRNA8220. (**e**,**f**) Apoptosis analysis of GMEC was detected with FCM after transfection with PCD5-circRNA8220 or si-circRNA8220. (**g**,**h**) Protein levels of Bcl-2, Bax, caspase 3 and caspase 9 in GMEC after transfection with PCD5-circRNA8220 or si-circRNA8220. Protein levels were measured by WB densitometry was normalised to the β-actin density from the same lane. Data were expressed as the means ± SEM. ** *p* < 0.01, * *p* < 0.05.

The effect of circRNA8220 on proliferation was checked by CCK8 and Edu assay in GMECs. The results of CCK8 showed that circRNA8220 promoted cell proliferation the result was recovered after co-transfection of pCD5-circRNA8220 with miR-8516 mimic (Figure 5a). Knockdown circRNA8220 inhibited cell proliferation (Figure 5b). The Edu results showed that circRNA8220 promoted cell proliferation, whereas si-circRNA8220 inhibited cell proliferation. The results were recovered after co-transfection of pCD5-circRNA8220 or si-circRNA8220 with miR-8516 mimic or miR-8516 inhibitor compared with transfection with pCD5-circRNA8220 or si-circRNA8220 (Figure 5c,d).

Apoptosis ratio was tested after transfection of pCD5-circRNA8220 or si-circRNA8220 by Annexin V-FITC/PI staining. The results showed that circRNA8220 inhibited the apoptosis of GMECs (Figure 5e), si-circRNA8220 induced the apoptosis of GMECs and the rate of apoptosis was decreased when si-circRNA8220 and miR-8516 inhibitors were co-transfected into GMECs (Figure 5f). The protein levels of Bax, caspase 3 and caspase 9 were up- or down-regulated after transfection with si-circRNA8220 or pCD5-circRNA8220 (Figure 5g,h).

### 3.6. STC2 Enhanced Synthesis of β-Casein and Triglycerides in GMECs

**Figure 6:**
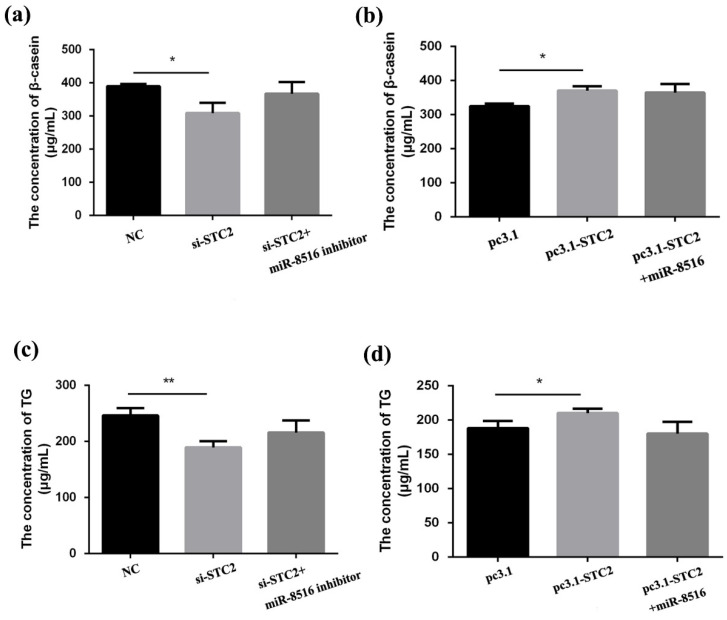
STC2 enhanced the synthesis of β-casein and triglycerides in GMEC. (**a**,**b**,**c**,**d**) Secretion of β-casein and triglycerides (TG) after transfection with pc3.1-STC2 or si-STC2 was measured by enzyme-linked immunosorbent assay kit. ** *p* < 0.01, * *p* < 0.05.

The concentration of β-casein and triglycerides were tested by ELISA KIT after 24 h transfection with STC2-siRNA or pcDNA3.1-STC2 to explore the effects of *STC2* on milk production in GMECs. The results displayed that production of β-casein and triglycerides was restrained after transfection of STC2-siRNA in GMECs (Figure 6a,c). Overexpression of *STC2* strengthened the concentration of β-casein and triglycerides (Figure 6b,d).

### 3.7. Ability of circRNA8220 to Promote Cell Synthesis β-Casein and Triglycerides Was Consistent with That of STC2

**Figure 7:**
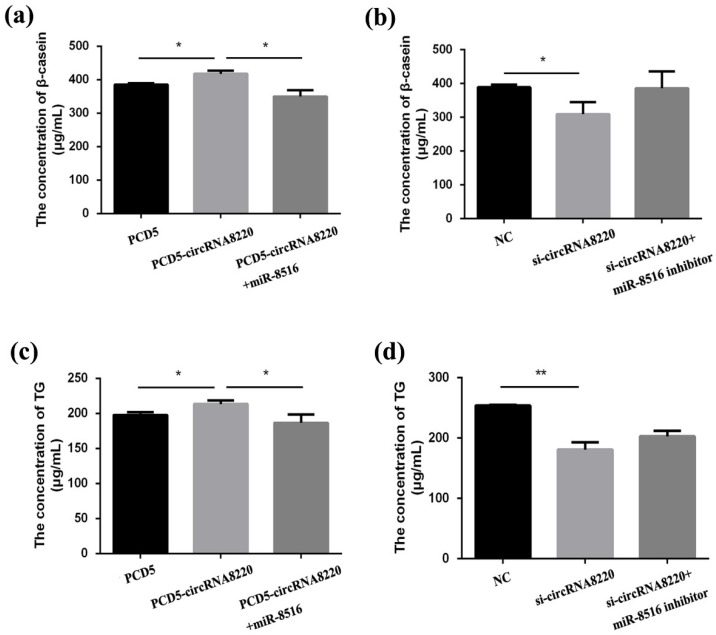
circRNA8220 enhanced the synthesis of β-casein and triglycerides in GMEC. (**a**–**d**) Secretion of β-casein and triglycerides (TG) after transfected with PCD5-circRNA8220 or si-circRNA8220 was measured by enzyme-linked immunosorbent assay kit. ** *p* < 0.01, * *p* < 0.05.

The concentrations of β-casein and triglycerides were also tested by ELISA KIT. Overexpression of circRNA8220 enhanced the synthesis ability of β-casein and triglycerides in GMECs (Figure 7a,c). Knockdown of circRNA8220 decreased the synthesis ability of β-casein and triglycerides in GMECs (Figure 7b,d). The result could be recovered after co-transfection of pcDNA2.1-circRNA8220 with miR-8516 mimic. Thus, circRNA8220 might facilitate the synthesis of β-casein and triglycerides through miR-8516-*STC2* pathways.

### 3.8. CircRNA8220 and STC2 Activated Ras/MEK/ERK Signaling Pathways in GMECs

**Figure 8:**
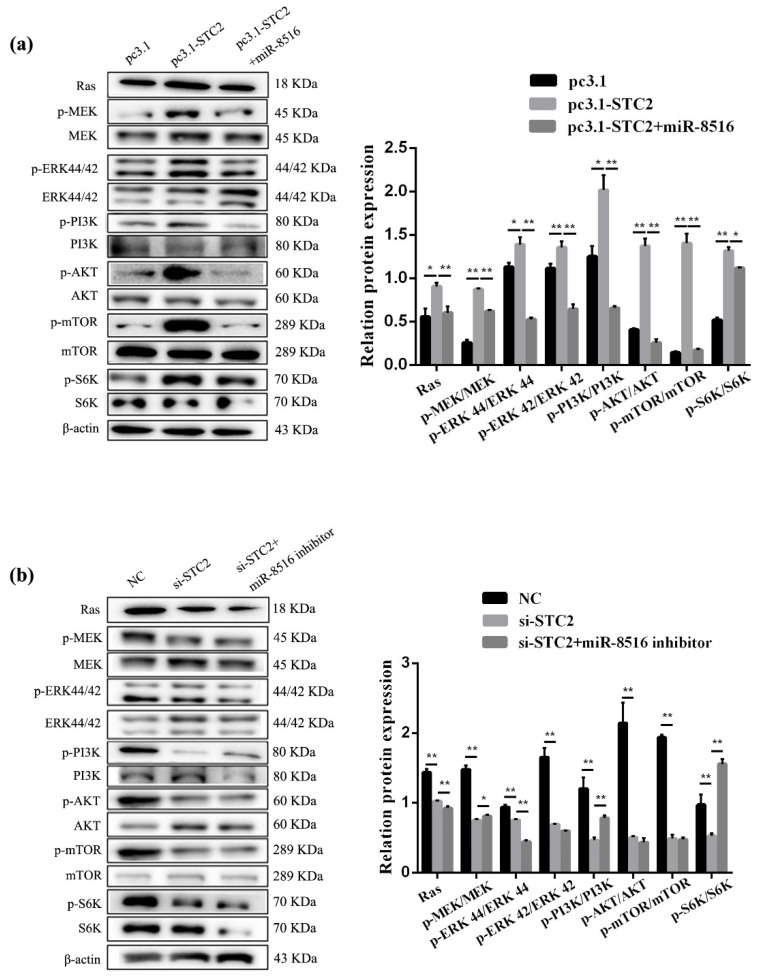
STC2 activated the signaling pathways of Ras/MEK/ERK and PI3K/AKT/mTOR in GMEC. (**a**,**b**) Western blot analysis was performed to detect the protein expression of Ras, p-MEK, MEK, p-ERK44/42, ERK44/42, p-PI3K, PI3K, p-AKT, AKT, p-mTOR, mTOR, p-S6K, S6K and β-actin in GMEC after transfection with pc3.1-STC2 or si-STC2. Protein levels were measured by WB densitometry was normalised to the β-actin density from the same lane. Data were expressed as the means ± SEM. ** *p* < 0.01, * *p* < 0.05.

Ras/MEK/ERK signaling pathways were tested to explore the regulation mechanism of *STC2* on apoptosis and proliferation in GMECs. Thus, the protein expression of Ras and the phosphorylation of MEK and ERK1/2 were evaluated by Western blot. The protein expression of Ras and the phosphorylation of MEK and ERK1/2 increased after transfection with pcDNA3.1-STC2 (Figure 8a). Compared with pcDNA3.1-STC2, STC2-siRNA had the reverse results (Figure 8b).

**Figure 9:**
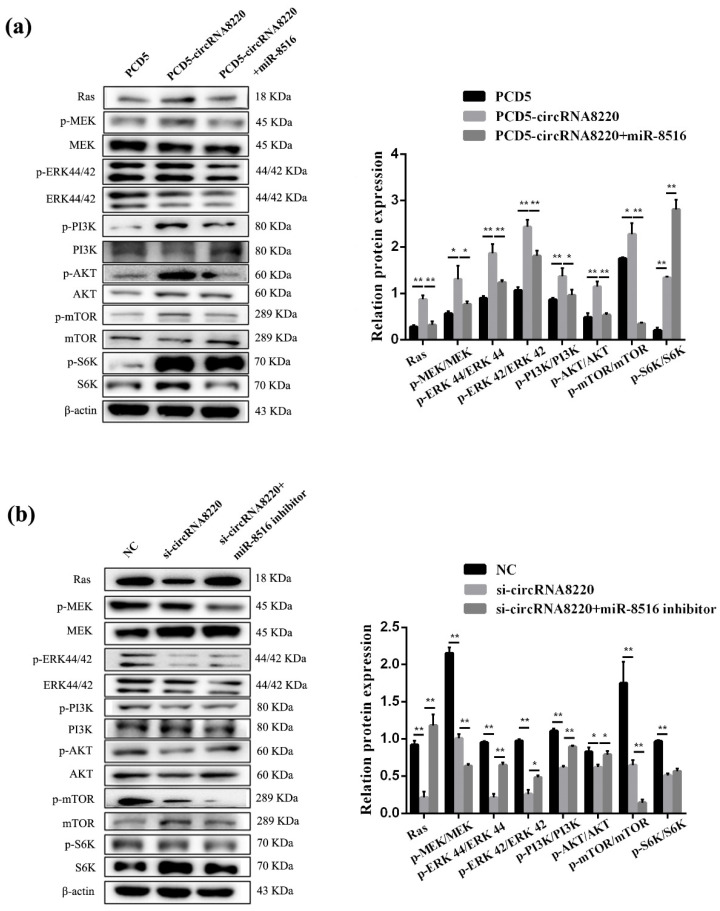
circRNA8220 activated the signaling pathways of Ras/MEK/ERK and PI3K/AKT/mTOR in GMEC. (**a**,**b**) Western blot analysis was performed to detect the protein expression of Ras, p-MEK, MEK, p-ERK44/42, ERK44/42, p-PI3K, PI3K, p-AKT, AKT, p-mTOR, mTOR, p-S6K, S6K and β-actin in GMEC after transfection with PCD5-circRNA8220 or si-circRNA8220. Protein levels were measured by WB densitometry was normalised to the β-actin density from the same lane. Data were expressed as the means ± SEM. ** *p* < 0.01, * *p* < 0.05.

Similarly, Ras/MEK/ERK signaling pathways were tested after transfection with si-circRNA8220 or pCD5-circRNA8220. The results showed that circRNA8220 increased the protein expression of Ras and the phosphorylation of MEK and ERK1/2 (Figure 9a). Knockdown circRNA8220 decreased the protein expression of Ras and the phosphorylation of MEK and ERK1/2 (Figure 9b). The effects of circRNA8220 on Ras/MEK/ERK signaling pathways were consistent with those of *STC2*. Moreover, circRNA8220 could positively regulate *STC2* in GMECs. These results showed that the protein levels of Bax, Bcl-2, caspase 3 and caspase 9 were consistent with those of *STC2* (Figure 5g,h).

### 3.9. CircRNA8220 and STC2 Activated PI3K/AKT/mTOR Signaling Pathways in GMECs

The molecular mechanism of *STC2* affecting milk synthesis was explored in GMECs. The phosphorylation levels of PI3K/AKT/mTOR signal pathways after transfection with pcDNA3.1-STC2 or si-STC2 were detected by Western blot. The results showed that high expression of *STC2* increased the ratio of p-PI3K/PI3K, p-AKT/AKT, p-mTOR/mTOR and p-S6K/S6K (Figure 8a) the phosphorylation levels of PI3K/AKT/mTOR/S6K signal pathway decreased when the expression of *STC2* was down-regulated in GMECs (Figure 8b). In the meantime, STC2-siRNA + miR-8516 inhibitor and pcDNA3.1-STC2 + miR-8516 mimic could more or less recover to the level of the control group.

As mentioned above, the ELISA result of circRNA8220 was consistent with that of *STC2*. The phosphorylation levels of PI3K/AKT/mTOR signal pathway were detected in GMECs after transfection with pCD5-circRNA8220 or si-circRNA8220 by Western blot to explore whether the molecular mechanism of circRNA8220 on lactation was consistent with that of *STC2*. The results showed that the effect of circRNA8220 on PI3K/AKT/mTOR signal pathways was consistent with that of *STC2* miR-8516 mimic or miR-8516 inhibitor could weaken the effect of pCD5-circRNA8220 or si-circRNA8220 on the PI3K/AKT/mTOR signal pathways in GMECs (Figure 9a,b). The above-mentioned results suggested a positive regulatory relationship between circRNA8220 and *STC2*. In other words, circRNA8220 could control PI3K/AKT/mTOR signal pathways through miR-8516/*STC2*.

## 4. Discussion

Many studies have reported that circRNAs could function as miRNA sponges to adjust the expression of gene in many physiological processes, such as gastric cancer [19], development of endometrial receptivity [20]. Recent studies showed that miRNA, circRNA and lncRNAs could function as regulators in mammary epithelial cell and be helpful for lactation, such as BTAT017009.2-miR-21-3p-IGFBP5 play an important role in cells proliferation in bovine mammary gland epithelial cells [21]. circHIPK3 promotes proliferation of cow mammary epithelial cells [22]. However, studies on circRNA in the regulation of lactation in dairy goat are limited. In this study, circRNA8220 displayed a sponging effect for miR-8516 in GMECs there was a negative feedback relationship between circRNA8220 and miR-8516 in GMECs the molecular mechanism of the negative feedback relationship require further research.

The nutritional value of goat milk is related to the proportion of milk components [23]. Many studies have reported that the PI3K/AKT/mTOR/S6K pathway was related to the synthesis of β-casein and triglycerides [24,25,26], study showed that overexpression of menin caused significant suppression of factors involved in the mTOR pathway, as well as milk protein κ-casein [27]. In this study, the results from ELISA KIT showed that overexpression of circRNA8220 promoted the synthesis of β-casein and triglycerides in GMECs. The result was contrary when the level of circRNA8220 was knockdown. Interestingly, the effects of si-circRNA8220 or pCD5-circRNA8220 on lactation were partially weakened by miR-8516 inhibitor or miR-8516 mimic. The results of WB showed that overexpressed or knockdown circRNA8220 up- or down-regulated the phosphorylation levels of PI3K, AKT, mTOR and S6K. These results explained that circRNA8220 promoted synthesis of β-casein and triglycerides by sponging miR-8516 in GMECs via PI3K/AKT/mTOR/S6K pathway.

ERK pathway inhibited cell apoptosis in many kinds of cells. For example, SOCS-1 inhibited apoptosis in cardiac myocytes via ERK1/2 pathway activation [28] varicella–zoster virus ORF12 protein inhibited apoptosis by induced phosphorylation of ERK1/2 in melanoma cells [29]. Our results showed that circRNA8220 could activate Ras/MEK/ERK pathway by sponging miR-8516, these results were consistent with Edu, FCM and CCK8. It was known that the number and activity of mammary epithelial cells are closely related to lactation and play an important role in breast development [1]. The casepase family is the initiator and executor of cell apoptosis in mammals, among which, caspase-3 is the most crucial apoptotic protease in the downstream of the caspase cascade reaction [30]. Bcl-2 inhibits the activation of the upstream caspase protease by interfering with release of cytochrome c, result in inhibition cell apoptpsis [30]. As a composition of the ion channel on the mitochondrial membrane, Bax protein allows cytochrome c to pass through the mitochondrial membrane, activating caspase-9 further activating caspase-3, thus resulting in cell apoptosis [31]. Procaspase-9, an initiator caspase in the mitochondrial pathway, is recruited and activated by the apoptosome leading to downstream casepase-3 processing [32]. The protein levels of critical apoptotic genes, including Bcl-2 [33], Bax [34], caspase 9 [35] and caspase 3 [36], we researched to prove the effect of circRNA8220 on critical genes of apoptosis. Our results showed that circRNA8220 could promote cell proliferation and inhibit apoptosis by sponging miR-8516. This result was opposite that of knockdown circRNA8220 in GMECs. Recent studies have shown that the expression level of Bax or Bcl-2 was inhibited or promoted by ERK in GMECs [37,38]. Together we draw a conclusion that circRNA8220 could promote cell proliferation and inhibit apoptosis by sponging miR-8516 in GMECs via Ras/MEK/ERK pathway.

*STC2* promoted proliferation in many kinds of cells, for example, *STC2* promoted hepatocellular carcinoma proliferation in vitro [15] and hepatocellular carcinoma cells proliferation [39]. The results of dual luciferase analysis, RT-qPCR and WB further proved that *STC2* was a target gene of miR-8516. We predicted that *STC2* may be in the regulation network of circRNA8220/miR-8516. The results showed that circRNA8220 increased the mRNA and protein levels of *STC2*. On the contrary, miR-8516 mimics decreased the mRNA and protein levels of *STC2*, miR-8516 inhibitor increased the protein level of *STC2*. As expected, the effects of pCD5-circRNA8220 and si-circRNA8220 were weakened by miR-8516 mimic and inhibitor. These results explained that circRNA8220 regulated the *STC2* by sponging miR-8516 as a ceRNA and proved the conjecture that *STC2* is in the regulation network of circRNA8220/miR-8516.

We speculated that circRNA8220 functioned as a miR-8516 sponge to promote proliferation, inhibit apoptosis and enhance the synthesis of β-casein and triglycerides via regulating *STC2* expression in vitro. The results showed that *STC2* promoted proliferation and inhibited apoptosis in GMECs in vitro. We also found that *STC2* enhanced the synthesis of β-casein and triglycerides in GMECs by the way of ELISA. All the results were consistent with those for circRNA8220. Therefore, *STC2* is in the regulation network of circRNA8220/miR-8516. Previous studies have shown that *STC2* could activate ERK in osteoblast [16,40]. ERK pathway inhibited cell apoptosis in many kinds of cells [28,29]. The results showed that overexpression of *STC2* enhanced the protein level of Ras and the phosphorylation level of MEK and ERK. This result was opposite that of knockdown *STC2* in GMECs. In addition, the protein levels of Bcl-2, Bax, caspase 9 and caspase 3 were explored in GMECs. As expected, the results showed that overexpression of *STC2* enhanced the protein level of Bcl-2 and inhibited the protein level of Bax, caspase 9 and caspase 3. These results were consistent with effect of circRNA8220 on Bcl-2, Bax, caspase 9 and caspase 3. In summary, circRNA8220 functioned as a miR-8516 sponge to promote proliferation and inhibit apoptosis via regulating *STC2* expression by Ras/MEK/ERK pathway in GMECs.

Similarly, we studied the regulatory mechanism of circRNA8220 and *STC2* on the effect of milk synthesis. The results of WB showed that *STC2* could activate PI3K/AKT/mTOR/S6K pathway. The results of *STC2* were consistent with those of circRNA8220 in PI3K/AKT/mTOR pathway. Overall, these results suggested that circRNA8220 functioned as a miR-8516 sponge to promote the synthesis of β-casein and triglycerides by regulating *STC2* expression via PI3K/AKT/mTOR pathway in GMECs.

## 5. Conclusions

**Figure A2:**
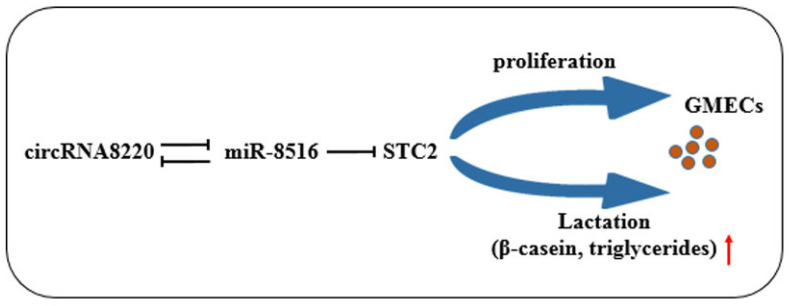
CircRNA8220 promote cell proliferation and enhance the synthesis of β-casein and triglycerides in GMEC in vitro.

In summary, this study researched the effect and regulatory mechanism of circRNA8220 and *STC2* on cell apoptosis, proliferation and lactation in GMECs in vitro. Therefore, a circRNA–miRNA–mRNA network is presented. We conclude that circRNA8220 as the sponge of miR-8516 can activate Ras/MEK/ERK pathway, promote proliferation and inhibit apoptosis by raising of *STC2*; it can also activate PI3K/AKT/mTOR pathway and enhance the synthesis of β-casein and triglycerides by up-regulating *STC2* in GMECs in vitro (Appendix A Figure A2).

## Figures and Tables

**Table 1:** Antibody used in this study.

Name	Manufacturer	Product Number
β-Actin	Beyotime, Shanghai, China	AA128
STC2	GeneTex, Alton Pkwylrvine, USA	GTX82231
Ras	Gene Tex, America	GTX132480
p-MEK1 (Ser298)	Abways, Shanghai, China	CY5854
MEK1	Abways, Shanghai, China	CY5168
p-ERK1/2 (Thr202/Tyr204)	Abways Shanghai, China	CY5277
ERK1/2	Abways, Shanghai, China	CY5487
p-PI3K (Tyr607)	Abways, Shanghai, China	CY6427
PI3K	Abways, Shanghai, China	CY6915
p-AKT (Ser473)	Cell Signaling, America	#9271
AKT	Cell Signaling, America	#9272
p-mTOR	Abways, Shanghai, China	CY6571
mTOR	Abways, Shanghai, China	CY5306
p-S6K (Ser424)	Abways, Shanghai, China	CY5261
S6K	Abways, Shanghai, China	CY5365
Bax	Abways, Shanghai, China	CY5059
Bcl-2	Abways, Shanghai, China	CY5032
Caspase 3	Cell Signaling, America	#9662
Caspase 9	Abways, Shanghai, China	CY5682
HRP-labeled Goat Anti-Rabbit IgG (H + L)	Beyotime, Shanghai, China	A0208
HRP-labeled Goat Anti-Mouse IgG (H + L)	Beyotime, Shanghai, China	A0216

## Abbreviations

circRNA	Circular RNA
GMECs	Goat mammary epithelial cells
STC2	Stanniocalcin 2
TG	Triglycerides
RNA	Ribonucleic
mRNA	Messenger RNA
miRNA	MicroRNA
UTR	Untranslated region
HEK293T	Human embryonic kidney cell line

## Appendix A

**Table A1:** RT-qPCR primers used in this study.

Gene	Genbank Accession No.	Primer Sequence (5′→3′)
β-actin	XM_018039831.1	F: GATCTGGCACCACACCTTCT
		R: GGGTCATCTTCTCACGGTTG
STC2 (qPCR)	XM_005694539.3	F: CGGAAGTGTCCAGCCATCAAGG
		R: CACAGGTCAGCAGCAGGTTCAC
STC2 (Check2)	/	F: CCCTCGAGTTGCCACCAGAGCAAAGCC
		R: TAAAGCGGCCGCTCTTGTCCCCCAGTGACGTG
Circ-8220 (qPCR)	/	F: GCCACAGCCTGGACATGAA
Circ-8220 (Check2)		R: CCTCTTGGTCACAGGGATGG
U6	/	F: cgCTCGAGTTCAAGATGCCCTGACCCCC
		R: atGCGGCCGCAGGTGAACTTCATGTCCAGGCT
miR-8516-Loop	/	F: CTCGCTTCGGCAGCACA
miR-449a		R: AACGCTTCACGAATTTGCGT
Reverse Primer	/	gtcgtatccagtgcagggtccgaggtattcgcactggatacgacGGCCTCCG
		gcgcgcGGCTGAGGGCAACGGAGGCC
	/	GTGCAGGGTCCGAGGT

Note: The characters with underscore were restriction enzyme cutting site of Xho I and Not I for constructing psiCHECK2.
